# Developing a Standardization Algorithm for Categorical Laboratory Tests for Clinical Big Data Research: Retrospective Study

**DOI:** 10.2196/14083

**Published:** 2019-08-29

**Authors:** Mina Kim, Soo-Yong Shin, Mira Kang, Byoung-Kee Yi, Dong Kyung Chang

**Affiliations:** 1 Department of Digital Health Samsung Advanced Institute for Health Sciences & Technology Sungkyunkwan University Seoul Republic of Korea; 2 Health Information and Strategy Center Samsung Medical Center Seoul Republic of Korea; 3 Center for Health Promotion Samsung Medical Center Sungkyunkwan University School of Medicine Seoul Republic of Korea; 4 Smart Healthcare & Device Research Center Samsung Medical Center Seoul Republic of Korea; 5 Division of Gastroenterology Department of Internal Medicine Samsung Medical Center, Sungkyunkwan University School of Medicine Seoul Republic of Korea

**Keywords:** standardization, electronic health records, data quality, data science

## Abstract

**Background:**

Data standardization is essential in electronic health records (EHRs) for both clinical practice and retrospective research. However, it is still not easy to standardize EHR data because of nonidentical duplicates, typographical errors, or inconsistencies. To overcome this drawback, standardization efforts have been undertaken for collecting data in a standardized format as well as for curating the stored data in EHRs. To perform clinical big data research, the stored data in EHR should be standardized, starting from laboratory results, given their importance. However, most of the previous efforts have been based on labor-intensive manual methods.

**Objective:**

We aimed to develop an automatic standardization method for eliminating the noises of categorical laboratory data, grouping, and mapping of cleaned data using standard terminology.

**Methods:**

We developed a method called standardization algorithm for laboratory test–categorical result (SALT-C) that can process categorical laboratory data, such as *pos +*, *250 4+ (urinalysis results)*, and *reddish (urinalysis color results)*. SALT-C consists of five steps. First, it applies data cleaning rules to categorical laboratory data. Second, it categorizes the cleaned data into 5 predefined groups (urine color, urine dipstick, blood type, presence-finding, and pathogenesis tests). Third, all data in each group are vectorized. Fourth, similarity is calculated between the vectors of data and those of each value in the predefined value sets. Finally, the value closest to the data is assigned.

**Results:**

The performance of SALT-C was validated using 59,213,696 data points (167,938 unique values) generated over 23 years from a tertiary hospital. Apart from the data whose original meaning could not be interpreted correctly (eg, ** and *_^*), SALT-C mapped unique raw data to the correct reference value for each group with accuracy of 97.6% (123/126; urine color tests), 97.5% (198/203; (urine dipstick tests), 95% (53/56; blood type tests), 99.68% (162,291/162,805; presence-finding tests), and 99.61% (4643/4661; pathogenesis tests).

**Conclusions:**

The proposed SALT-C successfully standardized the categorical laboratory test results with high reliability. SALT-C can be beneficial for clinical big data research by reducing laborious manual standardization efforts.

## Introduction

### Background

As the volume of digitized medical data generated from real-world clinical settings explosively increases owing to the wide adoption of electronic health records (EHRs), there are mounting expectations that such data offer an opportunity to find high-quality medical evidence and improve health-related decision making and patient outcomes [1-6]. EHR data collected during clinical care can support knowledge discovery that allows critical insights into clinical effectiveness, medical product safety surveillance in real-world settings, clinical quality, and patient safety interventions [1,7-12]. In recent years, interest is growing in conducting multi-institutional studies for earning strength in analysis using EHR data, such as the Observational Health Data Sciences and Informatics [13], National Patient-Centered Clinical Research Network [14], and Electronic Medical Records and Genomics network [15], by standardizing EHR data from multiple institutions [16-21].

Indeed, significant promising values are expected from using EHR. However, a substantial number of studies have mentioned that clinical data in EHR may not be of sufficient quality for research [22-27]. Compared with well-organized research cohorts or repositories, EHR systems are typically designed for hospital operations and patient care [28]. For example, a system may use local terminology that allows unmanaged synonyms and abbreviations. Thus, data of the same concept can be stored under different notations across different systems. Therefore, if these duplicate notations are not merged into a single concept, it can distort the results of a study. In addition, if local data are not mapped to standard terminologies, such as the systematized nomenclature of medicine (SNOMED) and logical observation identifiers names and codes (LOINC), performing multicenter research would require extensive labor.

Several EHR data standardization guidelines and tools for laboratory test name have been published [29-32], but there have been relatively few studies on data cleaning methodology for categorical laboratory data [33,34]. The label of laboratory results tends to be managed well for insurance claims, whereas laboratory results data, especially categorical results, are not well harmonized even in a single institution. Categorical laboratory results are usually written as free texts; different notations are used by departments or doctors, leading to significant data noise. Thus, harmonizing data becomes more challenging because it requires not only intensive labor but also clinical knowledge.

### Objectives

To resolve this drawback, there is a growing demand for data processing guidance and mapping tools for categorical laboratory data. In this study, we proposed a new automatic standardization algorithm for categorical laboratory results data, called standardization algorithm for laboratory test—categorical results (SALT-C). This algorithm was designed to help data curators by minimizing human intervention.

## Methods

### Overview

The original laboratory data used in this study are extracted from the clinical data warehouse (CDW) of Samsung Medical Center in Korea. The CDW contains deidentified clinical data of over 3,700,000 patients, including inpatient, outpatient, and emergency room patients, since 1994. The target dataset consists of 59,574,124 categorical laboratory results from 817 laboratory tests. This study focused on categorical data generated by machines; observation data, such as from health examination and allergy tests, were excluded even if sorted in categorical values.

### Defining the Categorical Laboratory Results Value Sets and Mapping Terminology

Before developing SALT-C, 5 value sets were predefined as a reference. The value sets were defined as follows. First, we analyzed the distribution of laboratory tests with their results. Second, from the most frequent laboratory tests, we defined the value set of each laboratory test by consulting physicians and referring to SNOMED value sets. Finally, we identified 5 common value sets by combing the value sets with similar values (Table 1). The value sets of the 5 categories were mapped into SNOMED identifiers, as SNOMED is the most popular international standard for clinical terminology. The mapping results are shown in Multimedia Appendix 1.

**Table 1:** Five common value sets.

Category	Value set
Urine color tests	Clear, cloudy, orange, purple, brown, green, blue, red, black, yellow, dark yellow, pink, turbid, milky white, amber, straw, colorless, bloody
Urine dipstick tests	Negative, normal, trace, +, ++, +++, ++++
Blood type tests	Rh+, Rh−, weak D, partial D, variant D, A, B, AB, O, cis-AB
Presence-finding tests	Positive, negative, weakly positive
Pathogenesis tests	Reactive, nonreactive, weakly reactive

### Developing the Automatic Standardizing Algorithm

**Figure 1:**
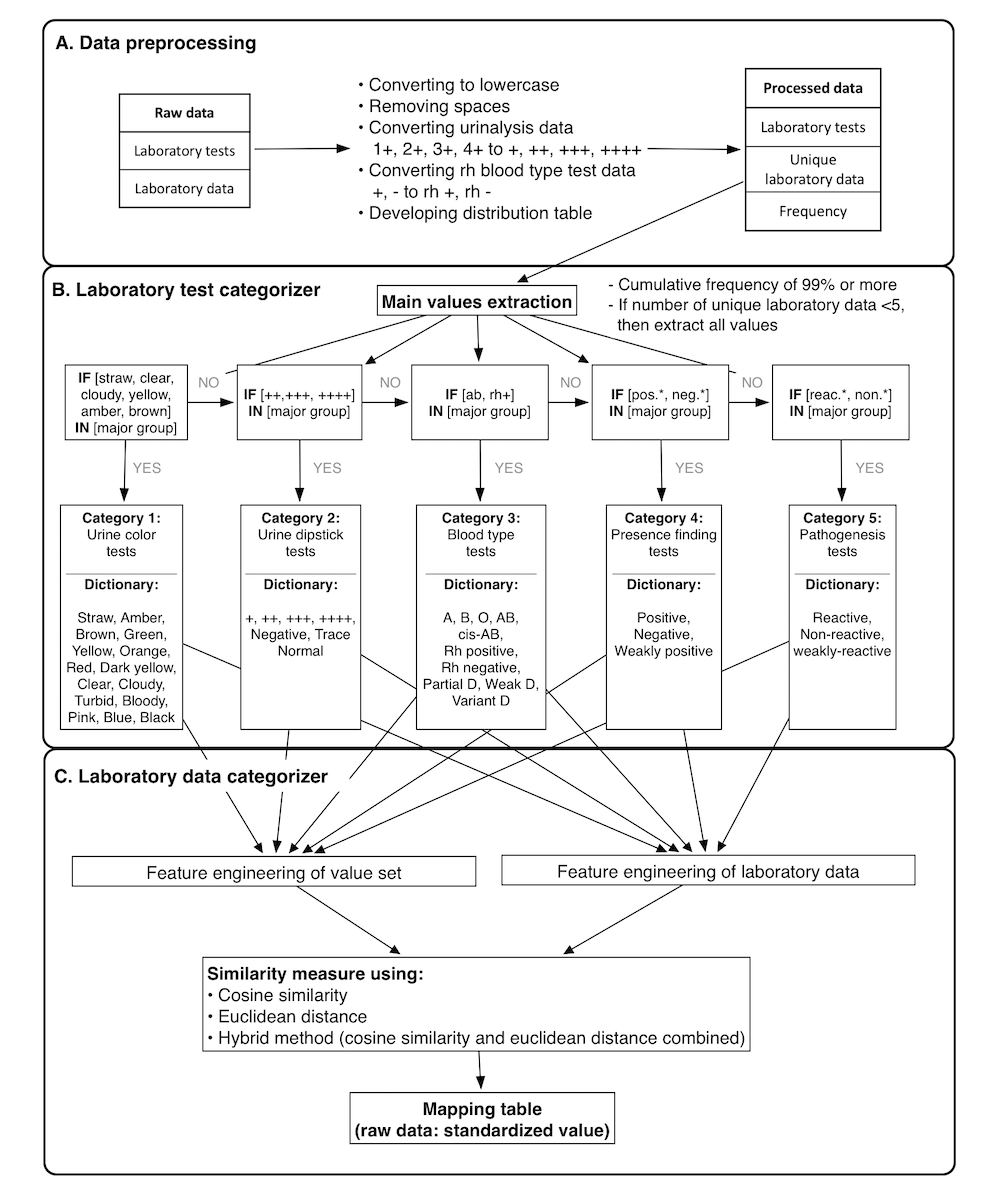
Process of the proposed standardization algorithm for laboratory tests—categorical results (SALT-C). neg: negative; pos: positive.

The overall procedure of SALT-C is described in Figure 1. Using the 5 common value sets developed in the previous step, we designed SALT-C to assign each laboratory test into one of the 5 value set groups (laboratory test categorizer), then assign the actual value to one of the standardized categorical items in the corresponding value set (laboratory data categorizer). Multimedia Appendix 2 demonstrates the entire process in detail. The following subsections will describe each method. SALT-C was written in Python. The source code of SALT-C can be downloaded using the GitHub link [35].

#### Data Extraction and Preprocessing

First, SALT-C extracts categorical laboratory data from a database or a comma-separated values format. Second, it preprocesses the extracted data with several methods: (1) applying the general data cleaning rules (ie, uppercase to lowercase and removing spaces from both sides), (2) correcting the abbreviation of *-* to *rh –* and *+* to *rh +* in *Rh blood type* laboratory data to distinguish it from the other *-* data of other laboratory tests, (3) formatting the urinalysis data. For example, results of urinalysis *4+* need to be converted into *++++*, which has SNOMED concept identifier 260350009.

#### Extraction of the Main Values From Each Laboratory Test

SALT-C creates a distribution table for each laboratory test to extract the representative values. The distribution table is implemented in the following order: classify the data for each laboratory test, calculate the frequency of the data, and organize them in descending order. After the creation of the distribution table, the main values of each test are extracted. Only the data with a cumulative frequency of 99.5% or more are extracted as main values.

In performing the experiments by changing the cumulative frequency, 99.5% seemed the most reasonable threshold, empirically. If there are less than 5 values in a laboratory test, then all the values are extracted as main values because the categorizer may not work properly if too few values are extracted as main values.

#### Laboratory Test Categorizer

Once the main values are extracted in the previous step, they are used to categorize the laboratory tests into 5 groups according to a rule-based categorizer, as in Figure 1. If one or more main values of a laboratory test are included in one of the predefined value sets, as in Table 1, the laboratory test is categorized into the corresponding category. The laboratory test categorizer proceeds in a specific order of test categories (urine color, urine dipstick, blood type, presence finding, and pathogenesis) until the laboratory test is assigned to a single category; most laboratory tests have *+* and *-* data as their main values and can be misclassified if they are not ordered.

We included the following when designing the laboratory test categorizer, to prevent laboratory tests from being assigned to incorrect categories: (1) correction of *-* and *+* data to *rh−* and *rh+* when they related to blood type tests, (2) classification of tests that have *++*, *+++*, and *++++* as main values in advance so that *+* data would not affect the subsequent classification, and (3) classification of blood type–related tests as a subsequent step; the remaining tests are classified into the presence-finding or pathogenesis category.

#### Character-Level Vectorization

**Figure 2:**
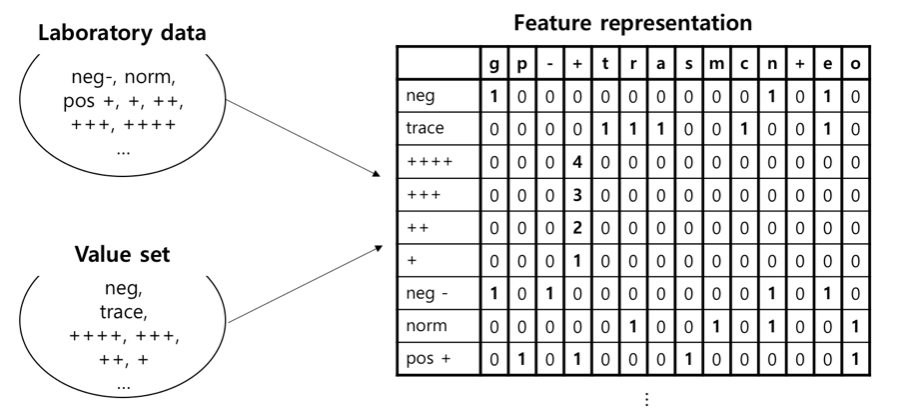
Character level vectorization. neg: negative; norm: normal; pos: positive.

In SALT-C, we choose the character-level vectorization to represent laboratory data. By vectorizing, only a limited number of alphabets of laboratory data are used, instead of laboratory test names. The scheme consists of alphabets (a-z) and special characters (-, _, and +). All data are represented as vectors with the number of characters corresponding to the scheme features. This process is described in Figure 2, with examples of the feature representation of urine dipstick tests category data.

#### Data Cleaning Using Similarity Measure

After all of the words are vectorized, a similarity score is calculated between a laboratory data point and each of the values in the standardized value set, and then the most similar value is selected. As a method of measuring similarity, we used and compared cosine similarity measure, Euclidean distance, and a hybrid method. The hybrid method was used to select the most similar value calculated by Euclidean distance when there are 2 or more same cosine similarity values.

### Manual Validation

We performed manual validation by adjudicating a total of 167,936 laboratory unique values that SALT-C predicted as labels. We examined the accuracy of the predicted labels calculated by the similarity measure. Three medical providers were recruited to manually verify data. Two of them examined the total data set and another person was involved to determine the final adjudication in the case of a discrepancy. The mean of the similarity scores for correct, incorrect, and unclassified data were identified.

## Results

### Dataset Descriptive Statistics

#### Distribution of Laboratory Tests

A total of 817 categorical laboratory tests and 59,574,124 test results were selected from the source database. The most frequent laboratory test was urinalysis (43,559,493, 73.12%), followed by hepatitis B blood (5,219,770, 8.76%), ABO/Rh blood type (3,261,992, 5.85%), hepatitis C blood (1,653,741, 2.77%), rapid plasma reagin (1,044,173, 1.75%), venereal disease research laboratory (551,980, 0.93%), *Treponema pallidum* latex agglutination (527,454, 0.89%), HIV (464,507, 0.73%), and hepatitis B blood test (1,653,741, 2.77%). Other tests had a rate of less than 0.5%. Additional results are described in Multimedia Appendix 3.

#### Distribution of Laboratory Data

**Figure 3:**
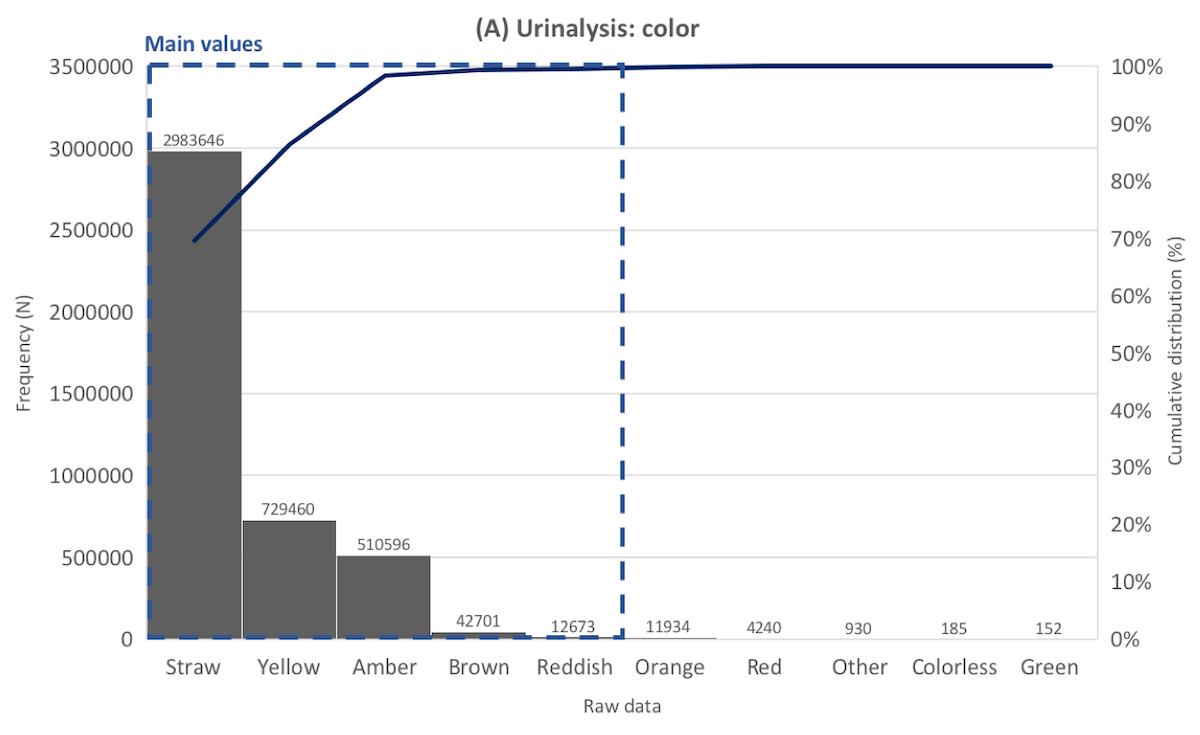
Distribution of laboratory tests data. Example laboratory test in the urine color tests category.

**Figure 4:**
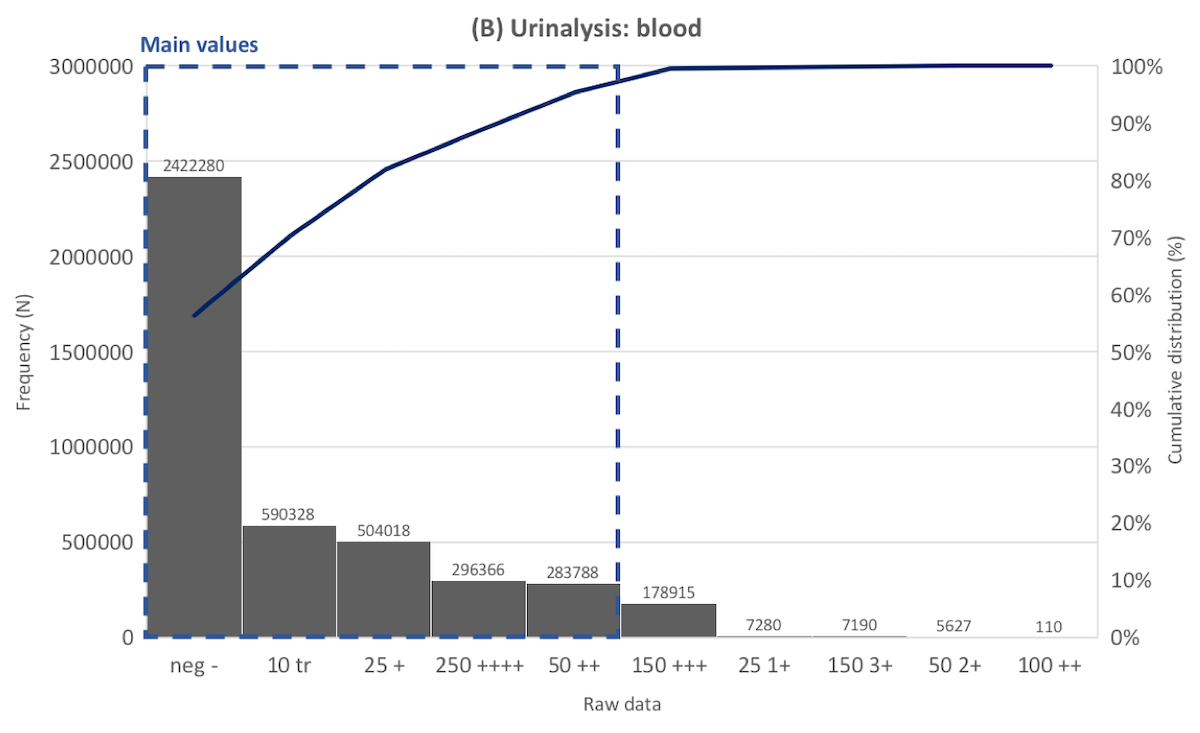
Distribution of laboratory tests data. Example laboratory test in the urine dipstick tests category.

**Figure 5:**
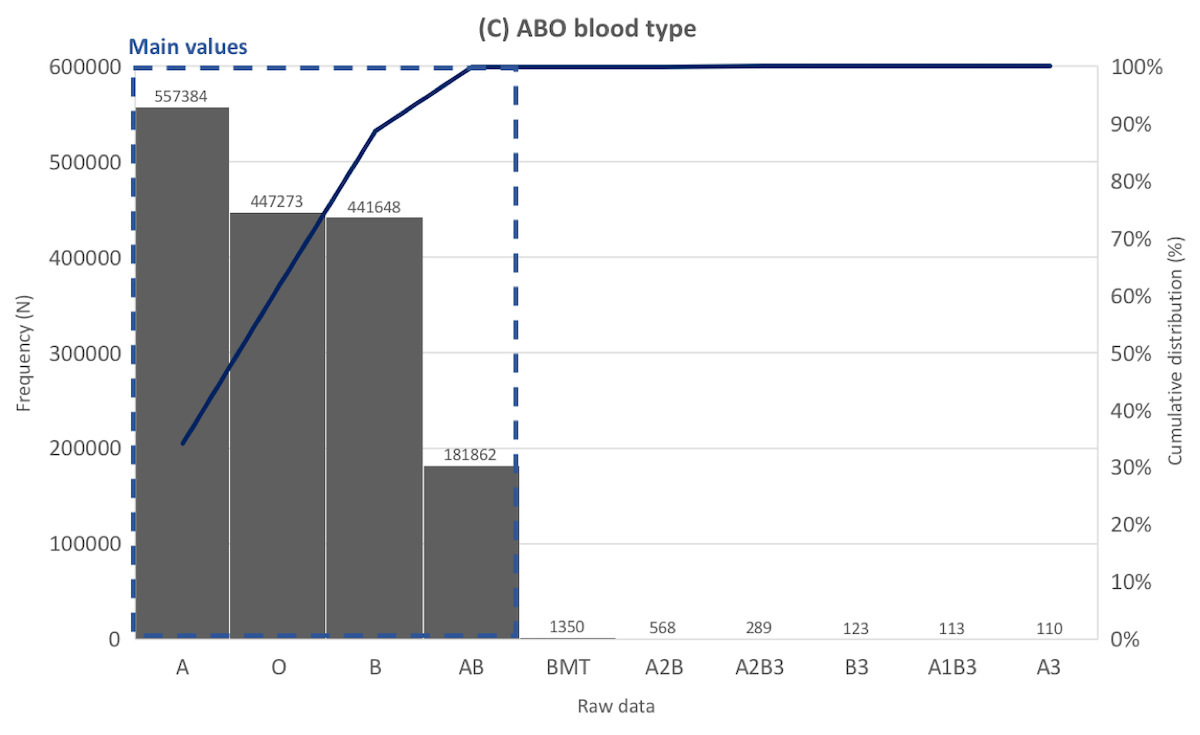
Distribution of laboratory tests data. Example laboratory test in the blood type tests category.

**Figure 6:**
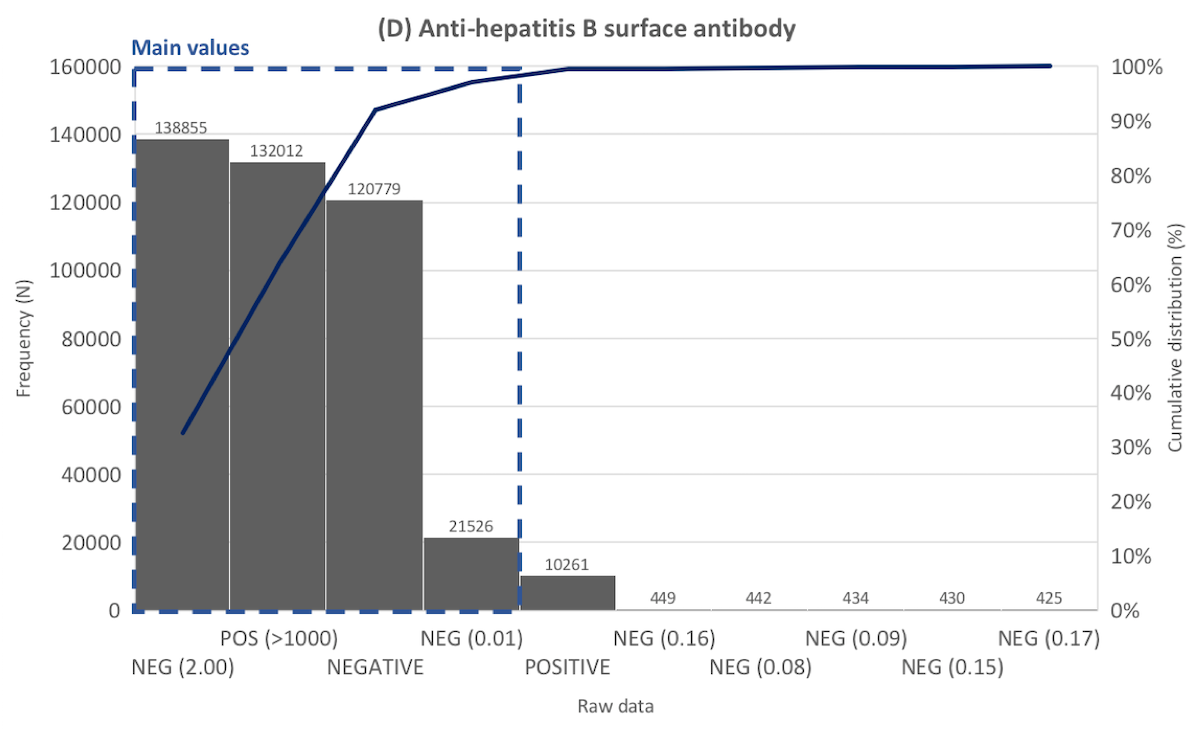
Distribution of laboratory tests data. Example laboratory test in the presence finding tests category.

**Figure 7:**
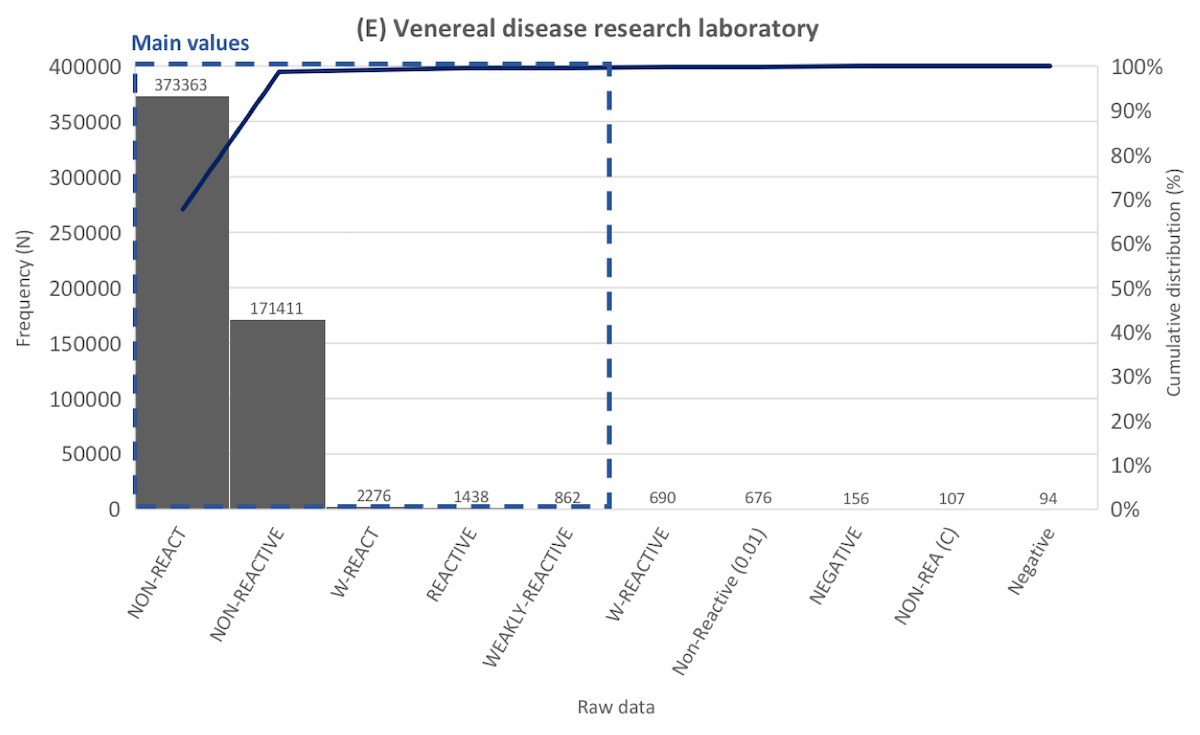
Distribution of laboratory tests data. Example laboratory test in the pathogenesis tests category.

Frequency distribution tables for laboratory data were created for the 817 laboratory tests. Representative distribution tables for each of the 5 categories are described in Figures 3-7 as histogram charts.

In the color test of urinalysis (Figure 3), there were 4,296,997 data points, of which 132 values were unique before preprocessing. The most common value was *Straw*, accounting for 69.43%, followed by *Yellow* (16.97%), and *Amber* (11.88%). Other data comprised less than 1%. *Straw*, *Yellow*, *Amber*, and *Brown* were extracted as main values according to the criterion that only data with a cumulative frequency of 99.5% or less are extracted as main values. The main values had various synonyms or typos and abbreviations. For example, the number of different notations that should be corrected as *Straw* was 151, for example, *Starw*, *]traw*, *Strow*, *Strwa*, *traw*, *]Straw*, and *steaw*.

As for the blood detection test in urinalysis (Figure 4), there were 4,296,700 data points, of which 235 values were unique before preprocessing. Various synonyms of the main values were identified, including typos and abbreviations. For example, *trace* had 29 such notation variations: *10 tr*, *25 tr*, *tr -*, *5 tr*, *tr*, *10 trace*, and *10 trt*. The most common value was *neg -*, accounting for 52.32%, followed by *10 tr* (13.73%), *25 +* (11.73%), *250 ++++* (6.89%), *50 ++* (6.60%), and *150 +++* (4.16%). Other data comprised less than 1%. Items *neg -*, *10 tr,* *25 +,* *250 ++++,* and *50 ++* were extracted as main values.

In ABO blood type laboratory tests (Figure 5), there were 1,630,995 data points, of which 53 values were unique before preprocessing. The most common value was *A*, accounting for 34.17%, followed by *O* (27.42%), *B* (27.08%), and *AB* (11.15%). Other data consisting of blood group variant (ie, *A*_1_, *A*_2_, *A*_3_, *A*_x_, *A*_m_, *A*_el_, and *A*_end_) comprised less than 1%. *A*, *O*, *B*, and *AB* were extracted as main values.

As the representative case of the presence-finding tests category, the antihepatitis B surface antibody laboratory test (Figure 6) had 1,190,631 data points, of which 56,134 were unique values before preprocessing. The most common value was *NEG (2.00)*, accounting for 11.66%, followed by *POS (>1000)* (11.09%), *NEGATIVE* (10.14%), and *NEG (0.01)* (1.81%). Other data comprised less than 1%. *NEG (2.00)*, *POS (>1000)*, *NEGATIVE*, and *NEG (0.01)* were extracted as main values. Laboratory tests belonging to this category usually had data composed of numbers and letters; thus, the number of unique values was far higher compared with other categories.

As the representative case of the pathogenesis tests category, the venereal disease research laboratory test had 551,980 data points, of which 130 were unique values before preprocessing (Figure 7). The most common value was *NON-REACT*, accounting for 67.64%, followed by *NON-REACTIVE* (31.05%). Other data comprised less than 1%. *NON-REACT*, *NON-REACTIVE*, *W-REACT*, *REACTIVE*, and *WEAKLY-REACTIVE* were extracted as main values.

### Categorization Results and 5 Common Value Sets

Overall, 5 categories and common value sets were created, and 480 laboratory tests were categorized into their corresponding group by the categorizer (Table 2). A total of 337 laboratory tests could not be classified. However, most of these uncategorized tests are not commonly used as these codes have been extinguished or temporarily issued for system testing. In addition, they only account for 0.61% of the raw data.

**Table 2:** Laboratory test categorization.

Category	Classified laboratory tests	
	Number	Representative laboratory tests
Urine color tests	2	Urinalysis: color, turbidity
Urine dipstick tests	14	Urinalysis: glucose, protein, ketones, hemoglobin, urobilinogen, bilirubin, leukocyte esterase
Blood type tests	3	Rh type, ABO group
Presence-finding tests	453	Hepatitis C virus antibody, Anti-HIV antibody, hepatitis B surface antigen, hepatitis B surface antibody, hepatitis B e-antigen, barbiturate screen, opiate screen, toxoplasma antibody, rubella antibody
Pathogenesis tests	8	Rapid plasma reagin, venereal disease research laboratory (VDRL), *Treponema pallidum* latex agglutination, VDRL (cerebrospinal fluid), *Treponema pallidum*

As shown in Table 2, 2 laboratory tests were categorized into the urine color tests category: one was the test for urine color and the other was the test for urine turbidity. The urine dipstick tests category included 2 sets of urinalysis tests, each consisting of 7 tests (glucose, protein, ketones, hemoglobin, urobilinogen, bilirubin, and leukocyte esterase) for checking the level of presence in urine. The blood type tests consisted of 2 tests related to blood type and 1 Rh type test. Most of the tests that have positive and negative data were categorized into the presence-finding tests. The pathogenesis tests category included 8 laboratory tests that were mostly related to sexually transmitted disease screening.

### Manual Validation of Similarity Measure Results

We examined 3 similarity measures, namely, cosine similarity, Euclidean distance, and hybrid method. For the mapping results of values, the hybrid method showed a 97.82% accuracy compared with cosine similarity (93.20%) and Euclidean distance (97.64%). For the mapping results of data, the hybrid method, with 99.99% accuracy, was also the most accurate compared with cosine similarity (93.78%) and Euclidean distance (99.96%), as shown in Table 3. Therefore, when using SALT-C with the hybrid method as a similarity measure, nearly all of the raw data were mapped to the target value. As for the unique laboratory values, the algorithm predicted labels with the following accuracy values: 97.62% (urine color tests), 97.54 (urine dipstick tests), 94.64% (blood type tests), 99.68% (presence-finding tests), and 99.61% (pathogenesis tests). Approximately 0.002% of the raw data that did not contain enough information for terminology mapping or were severely distorted were excluded from the analysis interpretation.

**Table 3:** Manual validation in unlabeled data.

Category		Cosine similarity		Euclidean distance		Hybrid method	
		Value	Data	Value	Data	Value	Data
**Urine color, n (%)**							
	Correct	123 (97.6)	8,592,841 (>99.99)	122 (96.8)	8,592,835 (0.49)	123 (97.6)	8,592,841 (>99.99)
	Incorrect	3 (2.4)	140 (<0.01)	4 (3.2)	146 (<0.01)	3 (2.4)	140 (<0.01)
**Urine dipstick, n (%)**							
	Correct	162 (79.8)	28,747,699 (93.96)	198 (97.5)	30,594,572 (>99.99)	198 (97.5)	30,594,572 (>99.99)
	Incorrect	41 (20.2)	1,846,897 (6.04)	5 (2.5)	24 (<0.01)	5 (2.5)	24 (<0.01)
**Blood type, n (%)**							
	Correct	50 (89)	3,261,963 (>99.99)	53 (95)	3,261,994 (>99.99)	53 (95)	3,261,994 (>99.99)
	Incorrect	6 (11)	44 (<0.01)	3 (5)	13 (<0.01)	3 (5)	13 (<0.01)
**Presence finding, n (%)**							
	Correct	162,291 (99.68)	14,788,631 (99.97)	162,296 (99.69)	14,788,663 (99.97)	162,291 (99.68)	14,788,631 (99.97)
	Incorrect	514 (0.32)	4021 (0.03)	509 (0.31)	3989 (0.03)	514 (0.32)	4021 (0.03)
**Pathogenesis, n (%)**							
	Correct	4643 (99.61)	1,944,729 (99.98)	4638 (99.51)	1,941,960 (99.84)	4643 (99.61)	1,944,729 (99.98)
	Incorrect	18 (0.39)	283 (0.01)	23 (0.49)	3052 (0.16)	18 (0.39)	283 (0.01)

## Discussion

### Principal Findings

The primary goal for this study was to find the way to efficiently map raw data to international standard terms. The first thing we did was to find standard value sets or code lists related to categorical laboratory test results. There are some value sets publicly available at SNOMED, LOINC, and Value Set Authority Center, but these were scattered, requiring an integrated dictionary to identify the spectrum of categorical laboratory data. Without an integrated reference dictionary, it is hard for researchers to convert their data into standard codes systemically, given that these data contain many synonyms, typos, and abbreviations. Such a situation has impeded easy organization and aggregation into standard terminology, as medical providers’ help is needed.

In this study, we identified 5 common value sets for categorical laboratory results by analyzing the distribution of laboratory tests with their results, by consulting with medical doctors, and by referring to laboratory tests’ SNOMED child codes. We found that 99.39% of the categorical test values fell into these value sets. As most of the categorical laboratory results were urinalysis data and data related to positive, negative, reactive, and nonreactive findings, and given that many researchers struggle with urinalysis data processing, we designed the value sets to handle as much urinalysis data as possible. The value sets developed in this study can be used for EHR interoperability, such as using Fast Health Interoperable Resources and Clinical Document Architecture. We continue to expand the values of value sets by applying SALT-C to several EHR databases internationally; furthermore, we are registering categorical laboratory value sets at Value Set Authority Center.

The laboratory data categorizer (Figure 1) measures the distance metrics between the standard item (eg, negative) and the laboratory test categorical values using a vector space model. We used the following method to increase computational efficiency and accuracy: (1) we only used the alphabets included in laboratory data, instead of alphabetical lists, as features and (2) we excluded duplicated characters in the standard term as much as possible. For example, *negative* and *positive* data were converted to *neg –* and *posi* to reduce similarity. We also attempted other string-matching methods, such as K-means clustering and Levenshtein distance; however, these 2 methods performed poorly. We demonstrated that the combination of cosine similarity and Euclidean distance method could give the best accuracy for laboratory test data, exceeding the performance of other measures. This hybrid model was complementary: the cosine similarity method selects the standard term with the most similar vector direction, and if the most similar vector direction is more than one, then the model adopts the closest value using the Euclidean distance method. For example, *+++ 6* data have the same cosine similarity scores for *+*, *++*, *+++*, and *++++*, respectively, but Euclidean distance indicates *+++* is the closest value. Usually, the cosine similarity is more accurate than Euclidean distance because it is less sensitive to the length or character order of terms; in some cases, the cosine similarity can be more accurate when combined with the Euclidean distance method. If there is a predefined code list table, it is more accurate to find the closest standard term by measuring the distance between the standard values and the data to be corrected; otherwise, the K-means method can be an alternative.

### Limitation

Our study has a number of limitations to be considered. First, we validated SALT-C through one institution; thus, it may not be generalizable to other institutions’ data. However, our manual validation of 167,936 data points proved the high performance of SALT-C. When it comes to applying this algorithm to other institutions, the framework suggested in this study can be used to process categorical laboratory data, and the accuracy of the algorithm can be increased by adding more values to the value sets. Second, we only targeted the diagnostic test results from devices, whereas data from observational health examinations, such as past history, family history, and manual allergy test results, were excluded. In the case of processing allergy test results, it is much more efficient to treat it as a regular expression method, so we did not include it in the algorithm. We believe that observational health examination data should be managed using a different table (ie, excluded from the laboratory test result table); the terms and structure of reporting these data are not well standardized, and as such, we were unable to include them in this study. Third, meaningless data or data that do not correspond to any values in the value sets were assigned standard values randomly. In this case, we suggest 2 solutions: (1) if the similarity scores measured by cosine similarity or Euclidean distance between the actual data and each of the standard values in the value set are the same, then these data need manual mapping; (2) as these data do not take up much of the total dataset, the rate of manual mapping will decrease by selecting the dataset corresponding to 95% of the cumulative frequency from the beginning. Fourth, we grouped blood group A variants such as *A*_1_, *A*_2_, *A*_3_, *A*_x_, *A*_m_, *A*_el_, and *A*_end_ into A, blood group B variants such as *B*_1_, *B*_2_, *B*_3_, *B*_x_, *B*_m_, *B*_el_, and *B*_end_ into B, and *cis*-AB into AB. However, it is more accurate to categorize blood group variants into subgroup [36,37]. We recommend modifying SALT-C algorithm depends on purpose of research regarding blood type.

### Future work

For the short-to-medium term, we plan to validate SALT-C algorithm with multiple institutions and add more values sets that covers more laboratory tests. In addition, as a series of SALT algorithm, we aim to develop standardization algorithm for laboratory test—allergy (SALT-A) that handles allergy data and standardization algorithm for laboratory test—blood culture (SALT-BC) that deals with semistructuralized blood culture results.

### Conclusions

We developed SALT-C, an algorithm that supports mapping of categorical laboratory data to the SNOMED-clinical terms (CT), and applied it to a large, long-period EHR system database. Previous studies on laboratory data processing have focused on the automatic mapping of laboratory test names or the standardization of numeric laboratory data [30-32,38]; however, we focused on categorical values of laboratory tests. Although SNOMED CT or LOINC standardize categorical laboratory test results, there is no widely accepted process of assigning standard codes to unstructured data fields.

There is an increasing need to aggregate and standardize EHR data to aid discovery of high-quality medical evidence and improve health-related decision making and patient outcomes. However, guidelines and automated methods for systemically converting disparate categorical laboratory data to standard terminology have been left to future work. The value sets and automated method suggested in this study may improve data interoperability and could be used for implementing standardized clinical data warehouse while reducing the manual effort of converting data. We plan to validate SALT-C through applying it at multisite institutions as well as expanding the value sets.

## Appendix

Multimedia Appendix 1 Mapping table of value sets.

Multimedia Appendix 2 The flow of SALT-C algorithm.

Multimedia Appendix 3 Distribution of categorical laboratory tests.

## Abbreviations

CDW: clinical data warehouse
CT: clinical terms
EHR: electronic health record
LOINC: logical observation identifiers names and codes
SALT-C: standardization algorithm for laboratory test—categorical results
SNOMED: systematized nomenclature of medicine
