# Supplementary material for: Developing a Standardization Algorithm for Categorical Laboratory Tests for Clinical Big Data Research: Retrospective Study
Source: JMIR Med Inform. 2019 Aug 29;7(3):e14083. doi: 10.2196/14083 (PMC6740165; doi:10.2196/14083)
Supplement: Multimedia Appendix 1 [file medinform_v7i3e14083_app1.pdf]

Supplementary table 1

Category	Classified laboratory tests		SNOMED mapping	
	Number	Laboratory tests	Value set	SNOMED Concept_id
**Urine color tests**	2	Urinalysis: Color, Turbidity	Clear	263707001
			Cloudy	81858005
			Orange color	371242008
			Purple color	371250004
			Brown color	371254008
			Green color	371246006
			Blue color	405738005
			Red color	371240000
			Black color	371252007
			Yellow color	371244009
			Dark yellow color	720001001
			Pink color	371243003
			Turbid	263906002
			Milky white	50935005
			Amber	None
			Straw	None
			Colorless	263716002
			Bloody	None
**Urine dipstick tests**	14	Urinalysis: Glucose, Albumin, Ketones, Blood, Urobilinogen, Bilirubin, Leukocyte Esterase, …	Trace	260405006
			+	441614007 260347006
			++	441517005 260348001
			+++	441521003 260349009
			++++	260350009
			Negative	Negative
**Blood type tests**	3	Rh type, ABO group	RhD positive	165747007
			RhD negative	165746003
			Weak D phenotype	89109006
			Blood group A	112144000
			Blood group B	112149005
			Blood group O	58460004
			Blood group AB	165743006
**Presence finding tests**	453	Anti - HCV antivody, Anti - HIV antibody, HBs Ag, HBs Ab, Hbe Ag, Barbiturate Screen, Opiate Screen,	Positive	10828004
			Negative	260385009
			Weakly positive	260408008
**Pathogeneses tests**	8	RPR type, VDRL, TPLA, VDRL(CSF), Treponema pallidum	Non-Reactive	131194007
			Weakly-reactive	117006002
			Reactive	11214006
