# Supplementary figures and images for: Developing a Standardization Algorithm for Categorical Laboratory Tests for Clinical Big Data Research: Retrospective Study
Source: JMIR Med Inform. 2019 Aug 29;7(3):e14083. doi: 10.2196/14083 (PMC6740165; doi:10.2196/14083)

**SALT-C algorithm:**  
Standardization Algorithm for  
Laboratory Test – Categorical Results)

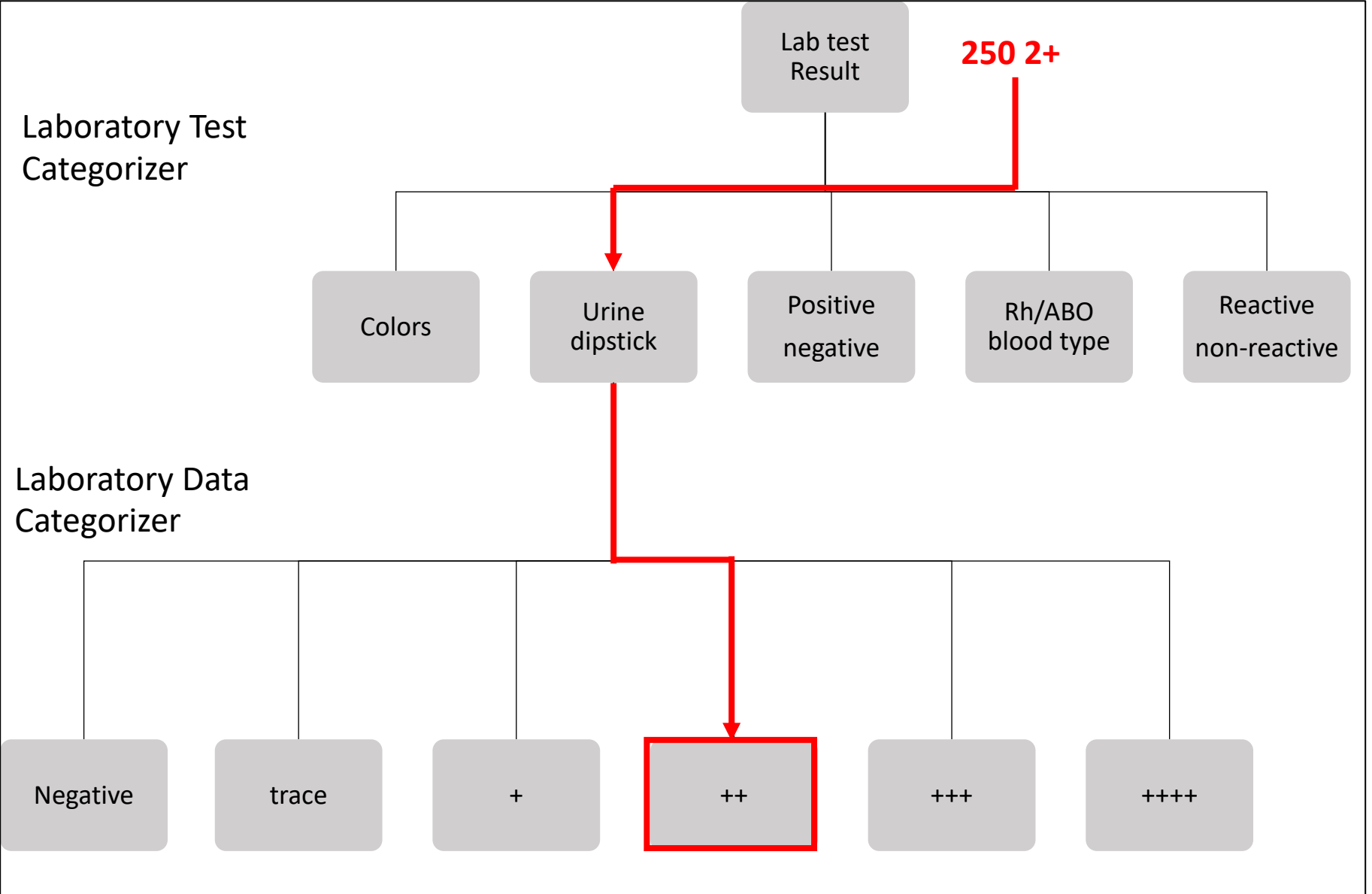

Supplement: Multimedia Appendix 2 [file medinform_v7i3e14083_app2.pdf]

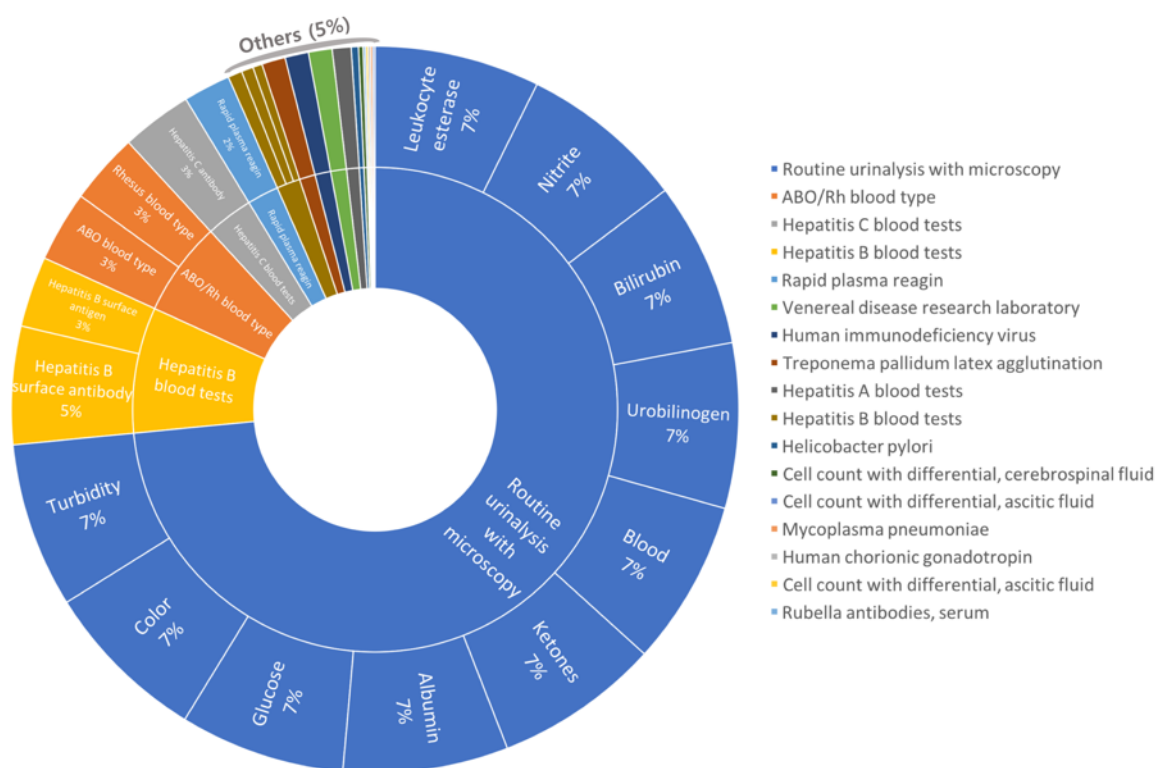

Supplement: Multimedia Appendix 3 [file medinform_v7i3e14083_app3.pdf]
